# Supplementary material for: XENet: Using a new graph convolution to accelerate the timeline for protein design on quantum computers
Source: PLoS Comput Biol. 2021 Sep 27;17(9):e1009037. doi: 10.1371/journal.pcbi.1009037 (PMC8496835; doi:10.1371/journal.pcbi.1009037)
Supplement: Attachment — Submitted filename: XENet_responses.pdf [file pcbi.1009037.s009.pdf]

Reviewer #1: *This is a very interesting paper. Unfortunately, the authors do not describe the quantum computing hardware, nor how the benchmarks were run on a quantum computer. What was this hardware, how many qubits, how much ECC, what QC architecture? How was noise handled? What about decoherence? The paper does not contain enough details.*

The reviewer rightly points out that full details of the quantum benchmark are needed. We have added a paragraph to the section titled “Benchmarking FixbbGCN Implementation On D-Wave Advantage Quantum Annealer” (formerly “Benchmarking FixbbGCN Implementation On Quantum Computer”) to provide details, for full reproducibility, of how we benchmarked using D-Wave’s 5,000-qubit system. However, since the FixbbGCN network rationally prunes rotamers to simplify a design problem, it could conceivably benefit any rotamer-optimization algorithm, quantum or classical. We have also added text at the start of this section clarifying that the particular benchmark that we performed is not the only conceivable quantum rotamer optimization algorithm that might benefit from FixbbGCN.

Second, running times could not be compared in the experimental designs.

Although running time could not be compared, it is currently limited by the coherence time of superconducting qubits, not by problem complexity. Classical memory consumption for the pre-computation of rotamer interaction energies, and consumption of qubits for the quantum optimization phase, do depend directly on problem complexity, however. We quantify the reduction in classical memory requirements, and have now added sentences to the section titled “Quantum FixbbGCN Benchmark” describing the reduction in qubit cost.

Third, the reductions in problem size, while interesting, are quite modest.

In our original submission, we did not properly convey the significance of a reduction in rotamer count. We have tried to make this clearer in our revised submission. The size of the solution space for a rotamer optimization problem involving N amino acid positions and D rotamers per position is D^N (*i.e.* the scaling is exponential). Reducing D, the number of rotamers per position, by a factor of M reduces the total number of possible solutions by a factor of M^N. For quantum algorithms that can scale exponentially with qubit count, this corresponds to an M-fold decrease in qubit requirements. For classical algorithms, this means a reduction in the size of search spaces by many orders of magnitude given even a modest reduction in rotamer count. We have added text to the ends of the sections titled “Quantum FixbbGCN-XENet Benchmark” and “Classical FixbbGCN-XENet Benchmark” making clearer the full impact of the reduction in rotamer counts that we were able to achieve.

Fourth, the Rosetta paradigm is notoriously difficult to parallelize beyond naive/embarrassing parallelism. It does not even use GPUs. A thorough discussion of other (more parallelizable) algorithmic paradigms for protein design (such as Hallen et al. J Comput Chem. 2018 Nov 15;39(30):2494-2507) is warranted.

The first and second paragraphs of the discussion have been considerably rewritten and expanded to review the many ways to solve rotamer optimization problems, including exact, parallelizable solvers like those implemented in Toulbar2 and Osprey; we now cite Hallen *et al*. *J. Comput Chem* 2018, Zhou *et al*. *Bioinformatics* 2014, and other relevant works. In our methods, we now mention that although we benchmarked with Rosetta, rotamer pruning should be of benefit to other methods as well. In our conclusion, we also mention that rational pruning of rotamers can benefit any classical rotamer optimization method (not just Rosetta).

Tom Schiex has showed and published that as the size of sequence increases, Rosetta almost never finds the GMEC, and that the number of sequences between the GMEC and the Rosetta answer is often in the tens of millions. QC could help with this if it is faster and more accurate. Can you show that QC addresses these flaws, improving not only speed, but accuracy?

We now discuss in the introduction the trade-off between finding the rotamer selection that globally optimizes the objective function versus rapidly sampling diverse low-energy states (which the heuristics in Rosetta tend to be useful for), and why diversity and exploration of the solution space near the optimum are desirable for a protein designer (especially given an imperfect scoring function). We cite Manon Ruffini and Thomas Schiex's recent work to adapt exact solvers to provide diverse near-optimal solutions rather than returning the single global optimum.

While we and many others are hopeful that quantum computing will provide a fast, favourably-scaling means of sampling from solution states near the global optimum, proving that is beyond the scope of this work. This work aims only to provide a means of mapping larger problems more relevant to the real world to nearer-term quantum hardware, so that future research can seek to answer that question for real-world protein design problems and not just for toy problems. We now mention this in the conclusion.

Reviewer #2: *This is an interesting utilization of graph neural networks to the problem of protein design with a view toward performing design on quantum computers. The description of the methods are well written and the direct comparison to other forms of graph networks are an important feature of the paper.*

I have only minor questions/comments which the authors could address:

1) it's odd to use the dihedral angles for the node and edge features in radians for phi,psi, CA1-CB1-CB2-CA2, etc but use sin and cos for the side-chain dihedral angles. It may not make much difference but what would happen if sin and cos were used for all the dihedral angles?

This is a great point and still an active area of research. We added this paragraph to the methods to address it:

Our model is inconsistent in handling dihedral angle values. Backbone torsion angles and trRosetta-inspired residue pair angles\cite{Yang2020} are represented as single values measured in radians, whereas side-chain chi angles are split into sine and cosine values. This decision was driven by the varying levels of confidence in a neural network's ability to interpret each metric. The trRosetta-inspired measurements were represented as single radian values in the original paper\cite{Yang2020}, which we felt no need to change. In our experience, neural networks do not benefit from splitting phi/psi angles into sine and cosine, however we do not have deep experience with modeling chi angles. In short, chi angles were split into sine and cosine for this project because we do not yet have confidence that neural networks can efficiently interpret them as single radian values, unlike the other angle features.

2) It is not quite true that different proteins in the top8000 set are distantly related to each other. In fact there are 1531 proteins in top8000 that have a homologue with greater than 50% sequence identity in top8000. Of the proteins used in this study, 1EDO has a 54% homologue in top8000 (3OSUB). Chain 1O4SA has a 40% homologue. This probably does not affect your results but you should be aware of it

Fair point. We removed the false claim about the top8000 ensuring diversity and instead just mentioned that these structures are high quality.

3) Proline does have two rotamers (chi1 = +30; chi1=-30). Why ignore that?

This is a great point. We updated this text to explain our reasoning:

For this project, rotamers from the 20 amino acids were binned into 54 categories. Alanine and Glycine each had their own bin due to their lack of meaningful χ1 attributes. Proline was also only assigned one bin despite having two χ1 rotamer wells \cite{Shapovalov2006}. The decision to simplify Proline's χ1 binning was driven by the high-risk, low-reward action of eliminating Proline rotamers from substitution rounds. Proline is valuable for design but is often represented by relatively few rotamers, so the reward of eliminating Proline is not great. For this reason, we wanted to let our neural network focus its resources on eliminating the other amino acids. The remaining 17 canonical amino acids had three bins each, which correspond to the three χ1 wells.

4) In Figure 4, the larger chains behave more predictably (a fall in energy at 60% rotamer coverage) but the smaller chains do not. Can you comment?

While it may just be error bars at plat, we do agree this is worth mentioning. We added this paragraph to the discussion section:

Fig 4 illustrates how larger problems generally behave more predictably than smaller problems. For the control (x-axis value = 1.0), the magnitude of the error is roughly 0.6\% for the smallest problem and roughly 0.2\% for the largest problem. Additionally, the impact of FixbbGCN appears to follow a more predictable pattern along the x-axis for larger sizes. One interpretation of this increasing predictability is that the larger proteins have more buried positions (unexposed to solvent). Buried positions are traditionally considered to be challenging to sample due to their inherently large number of neighbors. While this higher difficulty may exist, there may also be a higher degree of sampling predictability due to the confined local conformation of the buried pocket. We do not have enough evidence to do anything more than speculate at this time; future research would be required to dive into the intricacies of the rotamer substitution behavior.

(also list the PDB codes for the plots in the caption or above each plot).

We added this line to the caption:

Protein data bank codes from left-to-right are (top row) 1SFX, 1ECO, 1D4O and (bottom row) 1W2C, 1O4S, 1PJ5.

5) The node and edge features depend on the rotamer at each node and each pair of nodes. For instance, the side-chain/backbone or side-chain/side-chain hydrogen bonds must depend on the rotamer at each position (including chi2,chi3,chi4). So is the program run once for each rotamer of each residue type at each position so its features are determined? Then what are the values of the edge features, some of which depend on the rotamers/residue types at the neighboring nodes (up to N=30). Perhaps a diagram of the features and how they relate to the output (probability of a rotamer being included in a Rosetta design run) would be helpful.

This is a vitally important point for us to clarify. We added a "Model Input" subsection to the section on FixbbGCN, immediately prior to describing our node and edge features: FixbbGCN is intended to evaluate a rotamer substitution problem and make predictions about how the attempted solution to the problem will unfold. FixbbGCN does not analyze each rotamer in the set of candidates; instead, it analyzes the current state of the protein model prior to rotamer substitution. This limits the applicability of FixbbGCN to only be used with relatively refined protein models where the states before and after rotamer substitution are highly similar. We show this later in the paper by taking high-resolution crystal structures and re-optimizing their sequences. A counter-example would be **de novo** ***design on a generated backbone. FixbbGCN could still be used on that project but would require high-resolution model refinement beforehand.***

6) Very minor - I know the rotamer library is sort of part of the furniture in Rosetta :-) but it is a separate thing and Max's paper [Shapovalov and Dunbrack, 2011] can be cited when the rotamers are heavily discussed in a paper.

This is embarrassing on our part. That citation has been added.

Reviewer #3: *Summary: XENet presents an improved architecture for GNN-based networks informed by message-passing operations. The graph convolution contributions incorporate potentially asymmetric edge attributes representing physical or chemical features. XENet is applied as a preprocessing optimisation for a rotamer substitution protocol and then compared to existing CrystalConv and ECC architectures. In both classical and quantum computing benchmarks, XENet can reduce the problem size by a large factor before seeing a significant loss in data quality.*

Comments: The submission clearly defines its scope of current application to rotamer sample reduction and acknowledges previously established literature. Initial definitions are coherent, and data, figures and benchmarks are accessible to a non-specialist audience. XENet's contributions to message-passing GNNs and applications to optimising rotamer substitution are generally well-evidenced with potential improvements (see below).{

Specific concerns remain to be revised:

- The motivations for using an quantum annealer appear inadequately expressed in the text. While the paper acknowledges previous work by both the authors and others concerning quantum computing, an additional prefacing paragraph asserting the usefulness and advantages of near-term quantum computing may improve the overall contextualisation of the benchmarks and conclusions.

Good suggestion. The third paragraph of the introduction has been rewritten and expanded considerably to address this and to explain the reasons for seeking to map protein design to quantum computers.

- The cause of resource limitations for the fixbbGCN benchmarks could be expressed further. XENet is introduced as a suitable contribution to allow more significant protein design problems onto near-term quantum computers. However, the clarification within the quantum fixbbGCN benchmarks uses the smallest test case from the benchmark set. Is the limitation for the usable test case determined by any previous trials or estimations? If possible, perhaps a clarification on the differing tractability between quantum and classical approaches may help.

This is a critical limitation of this benchmark and we agree that it should be stressed more strongly. We touched up a few places in the introduction to explain why the quantum computers are so restrictive in general (low qubit resources). We also added a description in the methods to explain how we chose this size of benchmark and why the classical method does not have these limitations:

We found that any problem larger than this one would exceed the resources available to it for this benchmark. One relevant drawback of this quantum annealer is that all two-body energies must be exhaustively computed in advance; an $O(N^2)$ operation for a problem with N rotamers. This is another reason the classical benchmarks were able to test larger cases. Rosetta's simulated annealer is able to generate two-body energies on the fly, so the resource usage is linear with the number of considered substitution loops, which in turn is programmed to be linear with the number of rotamers.

Additionally, would it be feasible to estimate resource usage, perhaps by memory, for larger test cases within the benchmark set?

Offering any concrete estimation of hypothetical requirements is challenging and we are concerned that it would introduce more questions than answers. We do mention that the memory requirements are expected to scale quadratically with the number of rotamers in the problem. Our additions to the introduction also give insight into this relationship between problem size and resource requirements.

Other minor concerns for consideration:

- Definitions of (s) and (p) marking the same number of hidden layers and trainable parameters could be rephrased or moved, for the sake of clarity, to include CrystalConv as used in both Tables 1 and 2.

Good suggestion. We kept the (s) and (p) definitions in the XENet paragraph of the "Hidden Layer Sizes" section but altered the following paragraph to include:

Likewise, we benchmarked two CrystalConv models using the ***same two normalization techniques and similarly labeled them with (s) and (p).***

- Sparse grammatical errors are present in the author summary ("graphs data structures are ubiquitous [...]") and "Quantum FixbbGCN Benchmark" subsection ("[...] decrease the sizes our quantum annealing use cases.").

Good catch. We fixed those instances in this revision
